# Role of Glutaredoxin-1 and Glutathionylation in Cardiovascular Diseases

**DOI:** 10.3390/ijms21186803

**Published:** 2020-09-16

**Authors:** Mannix Burns, Syed Husain Mustafa Rizvi, Yuko Tsukahara, David R. Pimentel, Ivan Luptak, Naomi M. Hamburg, Reiko Matsui, Markus M. Bachschmid

**Affiliations:** 1 Vascular Biology Section, Whitaker Cardiovascular Institute, Boston University School of Medicine, 650 Albany St., Boston, MA 02118, USA; mannixb@bu.edu (M.B.); shmrizvi@bu.edu (S.H.M.R.); ytsuka@bu.edu (Y.T.); nhamburg@bu.edu (N.M.H.); bachschmid.markus@gmail.com (M.M.B.); 2 Cardiology, Whitaker Cardiovascular Institute, Boston University School of Medicine, 650 Albany St., Boston, MA 02118, USA; David.pimentel@bmc.org (D.R.P.); Ivan.Luptak@bmc.org (I.L.)

**Keywords:** glutathionylation, glutaredoxin, cardiovascular disease, redox signaling

## Abstract

Cardiovascular diseases are the leading cause of death worldwide, and as rates continue to increase, discovering mechanisms and therapeutic targets become increasingly important. An underlying cause of most cardiovascular diseases is believed to be excess reactive oxygen or nitrogen species. Glutathione, the most abundant cellular antioxidant, plays an important role in the body’s reaction to oxidative stress by forming reversible disulfide bridges with a variety of proteins, termed glutathionylation (GSylation). GSylation can alter the activity, function, and structure of proteins, making it a major regulator of cellular processes. Glutathione-protein mixed disulfide bonds are regulated by glutaredoxins (Glrxs), thioltransferase members of the thioredoxin family. Glrxs reduce GSylated proteins and make them available for another redox signaling cycle. Glrxs and GSylation play an important role in cardiovascular diseases, such as myocardial ischemia and reperfusion, cardiac hypertrophy, peripheral arterial disease, and atherosclerosis. This review primarily concerns the role of GSylation and Glrxs, particularly glutaredoxin-1 (Glrx), in cardiovascular diseases and the potential of Glrx as therapeutic agents.

## 1. Introduction

Over the past several decades, cardiovascular diseases (CVD) have been on the rise due to the heightened global prevalence of obesity and metabolic diseases [1,2]. Cardiovascular diseases account for the leading causes of death in the United States today and thus are a vital area of study [3,4]. As cardiovascular diseases are such a widespread issue, a profusion of research is being devoted to discovering the underlying causes and mechanisms to formulate potential treatments for many of these cardiovascular diseases. Unbiased omics approaches such as genetics, transcriptomics, metabolomics, and proteomics provide a deeper understanding of disease-associated pathways. CVDs are often associated with a perturbed redox environment [5,6,7], therefore studying the proteins involved in oxidant defense, repair, and redox signaling is such a vital aspect in the search for potential treatment of human diseases in the growing epidemic.

Glutathione (GSH) is an abundant small tripeptide functioning as a cellular antioxidant and redox buffer. The reversible addition of glutathione to protein cysteines forming a mixed-disulfide is referred to as glutathionylation (GSylation). GSylation is a physiological process that participates in redox signaling and protects protein cysteine thiols from irreversible oxidation during oxidative stress. Glutaredoxin-1 (Glrx) is an essential thioltransferase whose primary role is to remove protein GSylation [6]. While GSylation occurs transiently in physiology, a rise in GSylation is associated with various pathologies, perturbing normal redox signaling and cell function. Oxidative stress and inactivation of Glrx, in part, may contribute to the pathological protein GSylation.

Glrx plays an important role in the activity of an abundance of proteins and cellular processes by regulating the presence of these disulfide bonds, specifically through the reduction of GSH-protein disulfide bonds [8]. Glrx maintains the cellular redox homeostasis by regulating the GSylation of many vital proteins involved in signal transduction, such as phosphatases, kinases, and transcription factors. The roles of GSylation and Glrx depend on target molecules in the pathological condition. Glrx and GSylation have been implicated in a wide range of diseases, such as Parkinson’s [9,10,11], retinal degenerative disorders [12,13,14], non-alcoholic fatty liver disease [15,16,17], lung disease [18,19], and CVDs. Studies have shown that overexpressing Glrx may be an effective treatment for attenuating cardiac dysfunction in diabetic mice by maintaining redox homeostasis [20] as well as preventing cell death in rat cardiomyocytes under RNS stress [21].

In contrast, inhibition of Glrx is also vital for the revascularization of limbs following ischemia [22,23,24]. Research implicates Glrx controls fine tuning of redox signaling and may be a potential therapeutic target in a wide range of pathologies in which oxidative stress is involved. This review will focus on the GSylation and Glrx functions and targets in various types of CVD and discuss clinical applications.

## 2. Glutathion(GS)ylation

Glutathione (GSH), the tripeptide γ-L-glutamyl-L-cysteinyl-glycine, is one of the most abundant thiol redox-active molecules in the cell. Under normal physiological conditions, intracellular GSH concentrations range from 1 mM in most cell types to 10 mM in hepatocytes [8]. The concerted synthesis by glutathione synthetase and γ-glutamyl cysteine ligase provides the cell with GSH [25,26]. GSH is a vital cellular redox buffer and plays an essential role in combating oxidative stress, removing xenobiotics and toxins, synthesizing a variety of cellular signaling molecules [27,28], and regulating transcription factors. Various antioxidant enzymes, including glutathione peroxidases, glutaredoxins, and peroxiredoxins, use GSH as a thiol reductant to restore their enzymatic activities.

The sulfhydryl group (-SH) of protein cysteines can exist in a protonated (R-SH; thiol) or deprotonated state (R-S^−^; thiolate). The thiolate, as compared with the thiol, is chemically highly reactive and prone to oxidation. Various enzymes, including GAPDH and caspases use cysteine thiolates for biochemical catalysis in their active sites. Surrounding amino acids with basic or polar side chains such as histidine or serine, respectively, can stabilize the thiolate and markedly increase its chemical reactivity and sensitivity to oxidation. Exposure of the thiolate at the protein surface further renders it a likely target of redox modifications and regulation.

These reactive cysteine thiolates can exhibit a reactivity resembling that of a thiol-peroxidase and thus react with oxidants such as hydrogen peroxide. However, recent experiments suggest that hydrogen peroxide may not react directly; and peroxymonocarbonate is a reactive intermediate to oxidize cysteine of proteins [29]. The oxidation of the thiolate transiently produces sulfenic acid (R-SOH) that reacts with GSH leading to GSylation [30,31,32]. GSH acts as a thiol reductant and forms a mixed disulfide bond with the cysteine of the target protein, the first reduction step to prevent further irreversible protein thiol oxidation to sulfinic (-SO_2_H) and sulfonic acid (-SO_3_H). In a parallel NO-dependent pathway, these cysteines can be first nitros(yl)ated, and then GSylated [32,33,34].

GSH primarily functions as a redox buffer to maintain a reductive cellular environment for biochemical catalysis, a substrate for phase II detoxification reactions, and a reductant for cellular redox signaling and antioxidant defense. Cells experience oxidative stress when reactive oxygen species (ROS) such as hydrogen peroxide (H_2_O_2_) and superoxide, or reactive nitrogen species (RNS) including nitric oxide (NO), are generated in excess and overwhelm the cellular antioxidant system. Altered redox homeostasis, common to most CVDs, perturbs redox and nitric oxide signaling [35,36,37]. NO fluxes in the nanomolar range control vascular cell growth, migration, and adhesion, as well as vascular tone [38,39]. In contrast, higher NO concentrations generated by immune cells, such as macrophages, have cytotoxic and inflammatory effects [40].

**Figure 1:**
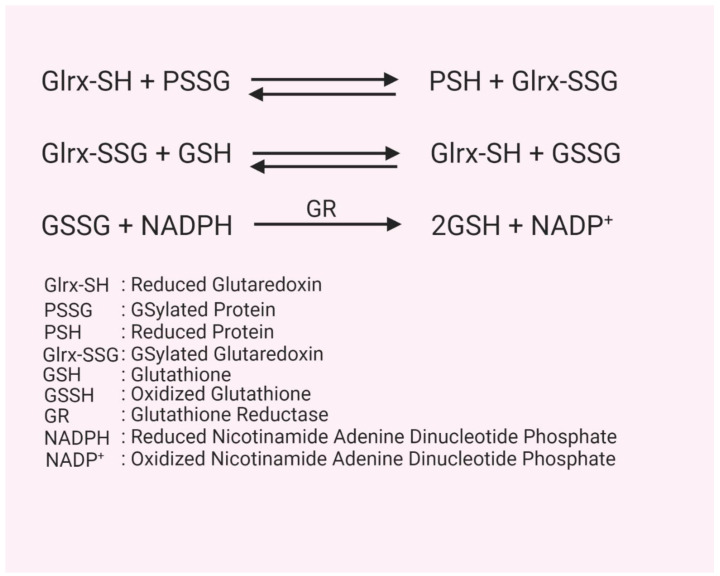
Glutaredoxin reaction. Figure represents steps of protein deGSylation by glutaredoxin-1 (Glrx) and regeneration of Glrx by glutathione (GSH).

GSH plays a vital role in antioxidant defense. For instance, the conversion of H_2_O_2_ to water is catalyzed by members of the glutathione peroxidase family [41]. In this reaction, the enzyme’s selenocysteine reacts with H_2_O_2_ to produce selenenic acid and water. In two reduction steps using GSH, the selenenic acid is converted back to selenocysteine, releasing oxidized glutathione (GSSG). Glutathione reductase, under the consumption of NADPH, reduces GSSG back to two GSH molecules [8]. Protein-bound GSH (PSSG) formation can significantly alter the structure, function, and activity of many proteins, including those involved in cell proliferation, cell death, and metabolism [8]. Protein-GSH mixed disulfide bonds are reduced by glutaredoxin (Glrx). The resulting GSylated Glrx (Glrx-SSG) is then reduced by GSH, forming GSSG and the reduced active Glrx [6] (Figure 1). The reductive cellular environment and GSH can also remove protein GSylation spontaneously without the enzymatic catalysis, but it proceeds at a markedly slower rate. Thus, Glrx deficient mice are not lethal, but their tissues have a significantly higher protein GSylation level under oxidative stress [42].

GSH is more abundant than oxidized GSSG under standard physiological conditions in the cell. In healthy organisms, a GSH:GSSG ratio of 100:1 is typical. This ratio can drop to below 10:1 under oxidative stress, therefore a low ratio of GSH to GSSG is widely considered a hallmark of oxidative stress and associated with various CVDs [43,44,45]. Since Glrx needs GSH as a cofactor, the low GSH environment may decrease Glrx activity, further increasing protein GSylation.

The transient nature and low abundance complicate the detection of physiological protein GSylation [46]. There are various methods to detect protein GSylation, each with its advantages and limitations. ^35^S-radiolabeling GSH (^35^S-GSH) is a highly sensitive and convenient method to detect GSylated proteins that requires prior inhibition of protein synthesis, followed by introducing radiolabeled ^35^S-cysteine into cells. Glutathione synthesis occurs independently from protein synthesis, thus, radiolabeled cysteine is mainly incorporated into glutathione. GSylated proteins can be isolated, separated on non-reducing gels, and detected by autoradiography or phosphorimaging. Loss of the radioactive signal after chemical or enzymatic reduction further confirms protein GSylation [47]. A similar method can be applied to detect GSylation using stable isotopes combined with mass spectrometry and proteomics [48].

Chemical or isotope labeled glutathione is an alternative method and can be introduced into cells as a cell-permeable ester. There is no need to inhibit protein synthesis, which can adversely affect cell physiology. In this method free thiols are blocked irreversibly by an alkylating agent. The modified thiols are then reduced by Glrx and labelled with biotin [49]. The biotinylated thiols undergo affinity purification with streptavidin and are analyzed by different proteomic approaches.

Antibody-based methods such as Western blot or immunoprecipitation allow direct detection of protein GSylation. However, commercial antibodies often lack sensitivity, produce high backgrounds, and work for select proteins only. Several other methods are also being used to detect GSylation via liquid chromatography-coupled mass spectrometry, (LC-MS)-based top-down proteomics [50], and ‘clickable’ GSH approach to detect GSylation [51]. As new technologies advance, overcoming these limitations may be possible in the near future.

## 3. Glutaredoxin Structure, Function, and Mechanism

Glutaredoxins (Glrxs) belong to the thioredoxin (Trx) protein family and catalyze a variety of processes including GSH-dependent redox reactions, the assembly of iron-sulfur (Fe-S) clusters, and redox signaling [52]. Glrxs contain a thioredoxin fold as an active site motif that is either Cys-X-X-Ser for the monothiol or Cys-X-X-Cys for the dithiol protein members. The thioredoxin fold is typically found in oxidoreductases and consists of a four-banded β-pleated sheet in between three alpha helices [53]. Glrxs have a cysteine in the N-terminal position of the active site, which is notably involved in the reduction of GSylated proteins (PSSGs).

There are four known members of the glutaredoxin family in mammals: glutaredoxin-1 (Glrx), glutaredoxin-2 (Glrx2), glutaredoxin-3 (Glrx3, also known as PICOT), and glutaredoxin-5 (Glrx5). These Glrxs differ in their functions, localization within the cell, substrate specificity, and amino acid sequence [54] (Table 1). Glrx and Glrx2 are dithiol, with their active site containing two cysteine residues for both Glrx (Cys-Pro-Tyr-Cys) and Glrx2 (Cys-Ser-Tyr-Cys), whereas Glrx3 [55] and Glrx5 [56] are monothiol, with only a single active site cysteine (Cys-Gly-Phe-Ser) [57]. Despite the difference in active sites, both types of Glrxs are able to bind GSH as a substrate and have varying degrees of oxidoreductase activity [52]. Glrx can perform both monothiol and dithiol reactions. In the dithiol reaction, Glrx launches a nucleophilic attack with its N-terminal active site cysteine onto a PSSG, forming a mixed disulfide. The available C-terminal active site cysteine of Glrx then attacks the mixed disulfide, forming oxidized glutaredoxin (Glrx-SSG) and releasing the reduced protein and a GSH molecule. Glrx-SSG is then reduced by two GSH molecules to reform active Glrx as well as GSSG. In the monothiol reaction, Glrx attacks the PSSG with its N-terminal active site cysteine resulting in the reduced protein and Glrx-SSG, which is then reduced by GSH to form Glrx and GSSG [6].

While it is only the primary function of Glrx, all four human Glrxs are capable of GSH-dependent oxidoreductase activity; they are able to reduce mixed disulfide bonds as noted earlier [52]. Glrx3 and Glrx5 notably reduce PSSGs at a much slower rate. Glrx2, Glrx3, and Glrx5 primarily function in iron-sulfur cluster assembly (2Fe-2S) and maintenance of iron homeostasis [64,65,66]. While not the focus of this review, the importance of these Glrxs and iron-sulfur clusters in many cellular processes such as DNA replication, DNA repair, transcription, and respiration should be noted [67,68]. Isoforms of Glrxs are described in detail elsewhere [69].

Glrx and Glrx3 are primarily contained in the cytoplasm, but Glrx may be present in the nucleus of some cell types as well as the mitochondrial intermembrane space [70], while Glrx2 and Glrx5 reside in the mitochondria. Glrx is considered as a main mammalian enzyme for de-GSylation and the multiple isoforms in the same location may ensure that redox regulation is tightly controlled. Glrx controls a wide variety of signaling pathways and cellular processes due to its role in regulating GSylation [8] in pathophysiological settings, including immune, reproductive, pulmonary, nervous, cancer, and cardiovascular diseases [52]. Glrx target proteins differ among organisms; they are vital components of important signaling pathways (Table 2).

## 4. Cardiovascular Disease

Protein GSylation has been implicated in CVD due to its importance in maintaining cardiovascular homeostasis. As the most abundant antioxidant in cells, GSH controls the regulation of many key biological processes and signaling pathways involved in CVD. Changes in protein GSylation levels and GSH:GSSG ratio are strongly correlated with the progression of CVDs, such as ischemia and reperfusion (IR), peripheral arterial disease, cardiac hypertrophy, and atherosclerosis.

### 4.1. Myocardial Ischemia and Reperfusion

Ischemia is known to increase protein GSylation levels, which stay elevated until the end of reperfusion [82]. Studies have focused on increasing Glrx in order to attenuate the heightened levels of GSylation during IR. Adenoviral Glrx gene therapy in type I diabetic mice protected hearts from IR injury, including decreased apoptosis of cardiomyocytes, reduced myocardial infarction size, and improved ventricular recovery [20]. Glrx controls apoptosis through modifications of proteins important in cell death pathways, such as activating Akt [79], NF-κB [24,72,83], and inhibiting Fas [84]. Akt activation results in the phosphorylation and deactivation of both FoxO and Ask-1, vital proteins involved in pro-apoptotic signaling pathways [20,79,85,86]. By inhibiting myocardial apoptotic signals, Glrx overexpression is able to attenuate infarction area and ventricular dysfunction in diabetic and non-diabetic mice [20,87]. GSylation of G-actin during IR decreased its polymerization rate and tropomyosin binding affinity, indicating that GSylation contributes to the decreased cardiac contractility associated with IR in rats [77,88].

GAPDH is one notable protein GSylated in high levels during ischemia and reperfusion (IR) [82]. A decrease in functional GAPDH due to GSylation increases apoptosis and inhibits glycolysis. We have shown oxidative or metabolic stress induces GAPDH GSylation in primary human aortic endothelial cells (HAEC). Gsylated-GAPDH is then translocated to the nucleus, where it interacts with histone deacetylating enzyme Sirtuin 1 (SirT1) and transfers GSylation to SirT1. This process, called trans-GSylation, inactivates SirT1 and results in the activation of apoptotic signaling by acetylation of the proapoptotic protein p53. Our results show that Glrx significantly de-GSylates GAPDH and protects HAEC’s from apoptosis by preventing the interaction of GAPDH with SirT1 [89]. This finding suggests that Glrx can also be utilized in favored redox signaling nodes like peroxiredoxin [90]. It is very likely that all peroxidatic cysteine enzymes act as signaling nodes and partner with very specific proteins in highly localized cellular subcompartments. Interestingly, GAPDH is GSylated in a cardiac rat IR model [82], implicating it as a possible signaling pathway leading to apoptosis in IR. The mechanism by which Glrx prevents GAPDH-SirT1 interaction through the regulation of GAPDH could have important implications in IR as well as other CVDs.

In contrast to other Glrx studies, Glrx KO mice with induced IR were shown to have similar vulnerability to acute oxidative damage as the control mice [42]. This indicates that cytosolic protein GSylation may not be as significant a cause of tissue injury in CVD as previously believed [42] or that other compensatory enzymes are upregulated to prevent progressive damage. Further, animal model research should be conducted to make more decisive conclusions on the ameliorating effects of Glrx on CVDs.

Hypoxia is a responsible factor for myocardial complications such as coronary heart disease. ROS generated during hypoxia/ischemia have adverse effects on metabolism, causing irreversible modifications of proteins, disrupt the redox state of the cells, and result in decline in cardiomyocytes contractility. Na,K ATPase maintains cardiomyocytes contractility by maintaining intracellular calcium levels. Irreversible modification of Na,K ATPase under hypoxic condition leads to cell death. Petrushanko et al. showed that acute hypoxia induces GSylation of Na,K-ATPase, inhibiting its activity and allowing the cell to avoid depletion of ATP before switching to anaerobic glycolysis. Thiol-containing compounds, including various GSH derivatives, significantly extend normal functioning of isolated rat cardiomyocytes under hypoxic conditions. Nitrosoglutathione (GSNO), which is known to induce GAPDH GSylation [33], was shown to promote faster recovery of isolated rat heart contractility by GSylation of Na,K ATPase following ischemia-reperfusion [91,92].

One prominent target of GSylation is nitric oxide synthase (NOS). When NOS is GSylated, it undergoes a reversible process called uncoupling in which its enzymatic activity converts from NO production to superoxide production, reacting with NO to generate more potent oxidants, such as peroxynitrite (ONOO^−^) [80]. Thus, NOS uncoupling increases oxidative stress conditions and is associated with decreased vascular endothelial cell function or caused endothelial dysfunction, which leads to a variety of CVDs, including IR and atherosclerosis [93,94]. Studies have found that Glrx reverses NOS uncoupling via de-GSylation in vitro. It was separately concluded the inhibition of Glrx increases NOS uncoupling, and Glrx overexpression attenuates NOS uncoupling, implicating Glrx as an important enzyme in the regulation of NOS [95]. These results indicate that Glrx could improve cardiovascular conditions aggravated by NOS uncoupling.

In contrast, GSylation of sarco/endoplasmic reticulum Ca^2+^ ATPase (SERCA) Cys674 by an acute oxidant stimulates the protein, resulting in decreased intracellular Ca^2+^ levels and vasorelaxation [81]. NO-induced endothelial cell migration and Ca^2+^ uptake depend on Cys674, and Glrx overexpression inhibits the process [96] and manifests anti-angiogenic features as mentioned later. However, irreversible oxidation of Cys674 in association with chronic oxidative stress caused degradation of SERCA protein in diabetic pig aorta [97]. These differences in functional effects likely reflect the individual pKa of the protein’s cysteine residue and, by extension, quantity and concentration of redox that is available to the protein.

### 4.2. Cardiac Hypertrophy

Cardiovascular hypertrophy is commonly associated with many CVDs, typically occurring in early phases. Cardiac hypertrophy is characterized by an increase in cardiomyocyte size in order to increase the function of the cardiac pump while decreasing the tension on the ventricular walls. Increased functioning capacity of the cardiac pump may be initially beneficial; however, it greatly increases the risk of heart failure, sudden death, and other heart conditions in the long term [98,99].

While many factors contribute to the onset of cardiac hypertrophy, of particular interest is the Raf/MEK/ERK pathway [100]. Stimulation of this pathway is believed to occur through mechanical stress and G-protein coupled receptors but was also shown to be dependent on GSylation of Ras in studies on rat myocytes in vitro [58]. GSylated Ras increased ERK activity and the synthesis of proteins that drive cardiac hypertrophy. The same study presented evidence that adenoviral overexpression of Glrx attenuated cardiac hypertrophy through reduction of Ras-SSG, thus preventing increased downstream protein synthesis.

Our lab demonstrated that Glrx KO mice fed a high fat high sucrose diet (HFHS) develop cardiometabolic dysfunction, leading to left ventricular hypertrophy and fibrosis, which contribute to diastolic dysfunction. Glrx deficiency likely causes impaired the metabolic flexibility (Osaki et al., in prep.).

Notably, the role of Glrx on cardiac hypertrophy depends on the pathological model. Angiotensin II-induced cardiovascular hypertrophy was attenuated in Glrx KO mice in association with lower oxidants generation in aortas [101], speculating NADPH oxidase activation, which is essential to angiotensin II signaling, might be impaired.

In addition to Glrx, Glrx2 and Glrx3 have also been shown to play a role in cardiac hypertrophy. Deletion of Glrx2 in mice hearts results in cardiac hypertrophy and fibrosis as well as hypertension [61], while Glrx3 KO in mice results in cardiac hypertrophy and heart failure [63]. Glrx3 KO mice were found to have significant left ventricular hypertrophy compared to their WT counterparts at 12 months of age. This is due to increased production of ROS in cardiomyocytes as well as Ca^2+^ dysregulation after Glrx3 KO [61]. In another study, Glrx3 overexpression in transgenic mice was found to inhibit cardiac hypertrophy. Glrx3 was postulated to disrupt interactions between muscle LIM protein (MLP) and calcineurin, which inhibits calcineurin-NFAT signaling, an important signaling pathway that promotes cardiomyocyte growth [102]. These studies exemplify that several Glrxs play roles in attenuating cardiac hypertrophy and will likely be dependent on protein interactions and sub-cellular localization.

### 4.3. Peripheral Arterial Disease

Glrx has been implicated in peripheral arterial disease and impaired vascularization in the limbs. In contrast to beneficial roles of increased Glrx in post-ischemic cardiac revascularization [87], Glrx overexpression inhibits angiogenesis in mouse hindlimb ischemia [24], while Glrx deletion in mice improves limb revascularization following ischemia [23]. Glrx inhibits angiogenesis through control of several target proteins, including tyrosine phosphatases, Rac1, HIF1-α, and NF-κB [22]. Glrx-induced Rac1 activation leads to inhibition of endothelial permeability [59] which may suppress endothelial cell migration. HIF-1α, a key angiogenic transcriptional factor, was stabilized by GSylation, further implicating the anti-angiogenic role of Glrx in hind limb ischemia [23]. The overexpression of Glrx is protective in the setting of cardiac ischemia but deleterious for ischemic limb, although both cases are associated with NF-κB activation. There are other cases showing the redox signaling in response to ischemia differs between the heart and muscles [22]. The main reason for this is probably that inhibiting apoptosis in acute ischemia is essential to myocardial survival, whereas angiogenesis potential is more important to ischemic limb recovery.

### 4.4. Atherosclerosis

Atherosclerosis can occur at large, medium, and small vessels leading to organ dysfunction due to ischemia, thrombotic phenomena, and tissue infarction [103]. Hyperlipidemia is an important risk factor for developing atherosclerosis [104,105]. Glrx KO has been shown to cause hyperlipidemia. Our group reported that Glrx KO mice develop hyperlipidemia, non-alcoholic fatty liver disease [17], and a cardiometabolic phenotype leading to diastolic dysfunction when fed a high fat diet (Osaki, et al. in prep).

Vascular permeability is postulated to be involved in giving rise to atherosclerosis and has been linked to a low GSH:GSSG ratio. It should be noted that GSylation and subsequent inactivation of Rac1 has been proved to play a role in vascular endothelial cell permeability. Glrx overexpression in high fat fed mice was shown to attenuate vascular permeability through deGSylation of endothelial cell proteins, particularly Rac1 [59].

One of the mechanisms behind atherosclerosis is damage to blood vessel wall endothelial cells, which are vital to upholding the integrity of the vascular wall as well as maintaining homeostasis [106,107]. This damage is often due to oxidative stress, which can induce apoptosis in endothelial cells. One of the most prominent apoptotic signaling proteins is Bim, a Bcl2 family protein that is most notably regulated by the transcription factor FoxO1, which is regulated by Akt through degradation after phosphorylation [108]. Furthermore, JNK (c-Jun *N*-terminal kinase), an important enzyme in stress response, has been shown to activate Bim through phosphorylation, promoting apoptotic signaling [109,110].

Endothelial cells, however, have protective mechanisms to combat oxidative stress in order to prevent apoptosis. Studies have shown that steady laminar blood flow reduces oxidative stress by inducing a reducing environment inside endothelial cells [111]. Laminar flow has also been shown to upregulate Glrx activity, which plays an important role in Akt activation [60]. Increased Glrx levels result in the prevention of apoptosis in endothelial cells by lowering Bim expression via Akt-FoxO1 signaling as well as by inhibiting JNK activation of Bim [89,112]. Additionally, as mentioned previously, Glrx may inhibit apoptosis signaling via NF-κB activation and Fas inhibition. This, along with new research indicating protein GSylation may be a major contributor to atherosclerosis, suggests Glrx may be useful as a therapeutic target in treating atherosclerosis [50].

### 4.5. Lipid and Glucose Metabolism

Glrx controls lipid metabolism as we show that Glrx KO mice develop fatty liver, hyperlipidemia, and obesity [17], which are risk factors for CVDs. A significant target molecule is sirtuin-1 (SirT1), an NAD+-dependent histone deacetylase, which is inactivated by GSylation [15]. The expression of lipid metabolism genes was upregulated in the Glrx KO livers, whereas adenoviral Glrx replenishment restored SirT1 activity, suppressed lipid metabolism genes and liver steatosis [17]. In humans, protein GSylation was increased, correlating with steatohepatitis, in pediatric patients with NAFLD [113]. Additionally, livers from patients diagnosed with hepatic steatosis preliminarily showed lower Glrx protein and increased GSylated protein expression [17].

Protein GSylation may stimulate adipogenesis by stabilizing the adipogenic transcription factor, CCAAT enhancer-binding protein (C/EBP) β in adipocytes. GSylation of C/EBP β inhibits its interaction with an E3 ubiquitin ligase, thus, inhibits its degradation and activates adipogenesis [114]. In a similar mechanism, GSylation stabilizes HIF-1α, and Glrx can inhibit its activation [23]. HIF-1α activates a variety of downstream genes relating to metabolism. HIF-1 induces glucose transporters and promotes glucose uptake, but also upregulates glycolytic enzymes and enhances anaerobic glycolysis, reducing mitochondrial metabolism. HIF-1 enhances lipogenesis by inducing genes involved fatty acid uptake and synthesis [115]. In mice, HIF-1α upregulation in adipocytes promotes obesity, glucose intolerance, inflammation, and fibrosis, while its deletion shows the opposite effects [116]. HIF-1α-dependent PPARγ activation causes cardiac steatosis and hypertrophy [117]. Taken together, Glrx-induced reversal of GSylation is indicative of a beneficial role in controlling lipid and glucose metabolism. In addition, Glrx stimulates insulin exocytosis in pancreatic β-cells [118].

GSylation of some molecules may not necessarily be harmful to the body. Oxidants activate 5′-adenosine monophosphate-activated protein kinase (AMPK) via GSylation [119,120], which could improve metabolic homeostasis. GSylation inhibits tumor suppressor p53 [121], which may inhibit cell death and protect hearts from ER stress [122]. However, diet-induced diabetes in Glrx KO mice enhanced pathological phenotype in the liver and the heart. We showed that inhibition of SirT1 by GSylation increased acetylated-p53, which can activate p53 and promote cell death [17]. There may be a sort of hierarchy in vivo that oxidants control signaling by GSylation.

The role of Glrx2 on metabolism is more complicated. Glrx2 KO mice worsened high-fat diet-induced insulin resistance and weight gain [123] and developed cardiac hypertrophy and hypertension with impaired mitochondrial ATP production [61]. However, heterozygous Glrx2+/− mice have attenuated high-fat diet-induced weight gain and hyperlipidemia [124]. These results likely are a result of the different mouse background used. In addition, neither of these studies used reconstitution of Glrx2 to confirm that this was the sole etiology of the phenomena.

## 5. Summary and Translational Application

Several clinical studies have investigated the correlation between GSH levels and a variety of CVDs. Decreased levels of GSH have been observed after myocardial infarction and stroke [125], type II diabetes [126], as well as various heart transplant surgeries [127]. Moreover, some studies observed that CVD is correlated with low ratios of GSH:GSSG, including in patients with hypertension [128], type II diabetes, or coronary heart disease [129]. A study involving 134 CVD patients and 435 healthy control subjects revealed that blood plasma GSH levels were significantly lower in the CVD patients. The relationship between low plasma GSH levels and increased risk for CVD was observed for a variety of CVD types [125]. Similar results were detected in patients with coronary heart disease in a study on 425 coronary heart disease patients and 225 healthy control subjects [129]. Another study observed that in comparison to healthy control individuals, patients with type II diabetes mellitus have a significant two-fold decrease in the GSH levels of both red blood cells and blood plasma, as well as a three-fold decrease in the GSH levels of monocytes [126]. Since Glrx uses GSH (as well as GSSG reductase) as a co-factor, lower GSH:GSSG may limit Glrx activity, resulting in effects similar to Glrx deficiency. For this reason, therapeutic Glrx administration may need concomitant GSH supply.

**Figure 2:**
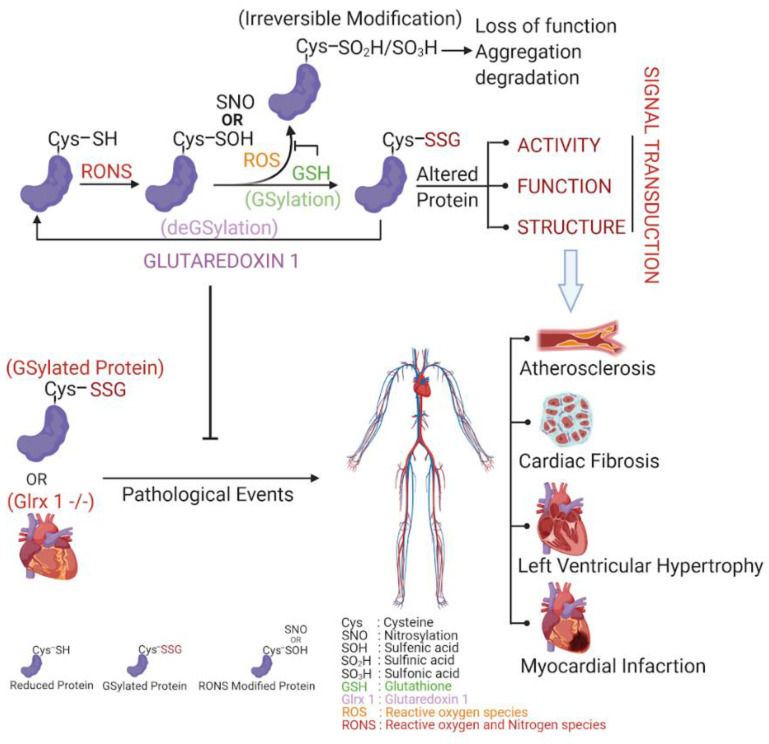
GSylation, deGSylation and its pathological events. Protein cysteines are readily oxidized by RONS as reversible S-OH or S-NO. While these modifications are unstable/short lived, they are prone to irreversible modifications like HSO_2_/HSO_3_. The cell’s reducing environment tries to protect these cysteines from irreversible modifications. GSH, being one of the most abundant reductants of cells, protects protein cysteines by making GSH adducts through a process termed GSylation. While GSylation can alter the function, structure, and activities of proteins which affects signal transduction, Glrx 1 can reduce proteins by specifically removing GSH adducts called deGSylation. Hence, GSH and Glrx 1 maintains GSylation and deGSylation homeostasis in cells. Accumulation of GSylated proteins due to altered cell signaling or Glrx 1 insufficiency could induce pathological events like atherosclerosis, cardiac fibrosis, left ventricular hypertrophy, and myocardial infarction, etc.

While not many studies have researched Glrxs in the human heart, one study has shown that low levels of Glrx2 appear to be correlated with myocardial infarction, left ventricular hypertrophy, as well as fibrosis [62]. Although there have been fewer studies in humans of Glrx than GSH, one notable study examined the localization of Glrx within human coronary arteries. It was discovered that in nonatherosclerotic coronary arteries, Glrx was expressed in smooth muscle cells, fibroblasts, and endothelial cells, whereas macrophages were observed to have highly expressed Glrx in atherosclerotic lesions. The same study concluded that the generation of ROS within cultured human coronary artery smooth muscle cells was correlated with increased Glrx expression, implicating that Glrx could play a role in atherosclerosis due to its antioxidant effect [130]. Clinical studies have also shown that basal plasma Glrx level is significantly lower in pre-cardiac surgery patients than in healthy control patients [131]. Decreased plasma Glrx in CVD patients could be due to the long-term increased level of oxidative stress associated with the pathology; however, the mechanism of decreased plasma Glrx in CVD patients is to be determined (Figure 2).

In summary, Glrx protects cardiomyocytes and endothelial cells from acute oxidative stress-induced apoptosis, and also may prevent cardiometabolic dysfunction, suggesting its beneficial effects and potential therapeutic use for CVDs. While there have not been clinical trials testing Glrx as a therapeutic agent in CVD, advances in biological technologies such as gene therapy [132] and gene editing (e.g., CRISPR) [133], as well clinical Glrx administration as therapy for lung fibrosis [18], indicate potential going forward [134]. However, Glrx controls the fine balance of redox homeostasis and likely regulates different signaling pathways depending on the pathology. Further studies are required before applying conclusions from mice to humans. As displayed in this review, an ever-growing body of work implicates Glrx as a promising avenue for further research in the field of cardiovascular biology.

## Figures and Tables

**Table 1:** Characteristics of human glutaredoxins. Glrx, glutaredoxin-1; Glrx2, glutaredoxin-2; Glrx3, glutaredoxin-3; Glrx5, glutaredoxin-5; C, cysteines; P, proline; F, phenylalanine; Y, tyrosine; S, serine; Da, daltons; PICOT, PKC-interacting cousin of thioredoxin.

Human Glutaredoxin	Reaction Mechanism	Primary Cellular Compartment	Primary Molecular Function	Aliases	Cardiovascular Pathologies	Active Site Motif	Mass (kDa)
Glrx	Monothiol or dithiol	Cytosol (Mitochondria, nucleus)	Glutathione oxidoreductase	Thioltransferase 1	Ischemia [20], cardiac hypertrophy [58], atherosclerosis [59,60], peripheral arterial disease [23,24]	CPYC	11.78
Glrx2	Monothiol or dithiol	Mitochondria	Iron-sulfur cluster assembly	Thioltransferase 2	Cardiac hypertrophy [61,62], myocardial infarction [62]	CSYC	18.05
Glrx3	Monothiol	Cytosol (nucleus)	Iron-sulfur cluster assembly	PICOT	Cardiac hypertrophy [63]	CGFS	37.43
Glrx5	Monothiol	Mitochondria	Iron-sulfur cluster assembly	N/A	N/A	CGFS	16.63

**Table 2:** Target proteins of glutaredoxin-1. GAPDH, glyceraldehyde 3-phosphate dehydrogenase; PTP1B, protein tyrosine phosphatase 1B; SERCA, sarco/endoplasmic reticulum Ca^2+^-ATPase.

Target Proteins of Glutaredoxin-1	Glutathionylation Induced Functional Changes	Identified Glutathionylated Cysteine Residues	Mass (kDa)
GAPDH [71]	Decreased oxidoreductase and transferase activity	150	36.05
NF-κB [72,73]	Transcriptional inactivation	179 (IKKβ), 62 (p50)	-
PTP1B [74]	Inactivation of protein tyrosine phosphatase activity	215	49.97
c-Jun [75]	Transcriptional inactivation	269	35.68
Rac-1 [59]	GTPase inactivation	81, 157	21.45
Creatine kinase [76]	Kinase and transferase inactivation	283	43.10
Actin [77]	Decreased polymerization rate and binding affinity	374	41.74
HIV-1 protease [78]	Decreased retroviral aspartyl protease activity	67, 95	10.73
Akt [79]	Inactivation of serine/threonine-protein kinase activity	297, 311	55.69
eNOS [80]	Uncoupling (conversion to superoxide production)	689, 908	131.12
Ras [58]	Increased GTPase activity	118	21.30
HIF-1α [23]	Stabilization and transcriptional activation	520	92.67
SirT1 [16]	Deacetylase inactivation	61, 318, 613	81.68
SERCA 2 [81]	ER Ca^2+^ ATPase activation	674	114.14

## Abbreviations

GSylation	glutathionylation
GSH	glutathione
CVD	cardiovascular disease
Glrx	glutaredoxin
